# The Emergence of Mesolithic Cemeteries in SW Europe: Insights from the El Collado (Oliva, Valencia, Spain) Radiocarbon Record

**DOI:** 10.1371/journal.pone.0115505

**Published:** 2015-01-28

**Authors:** Juan F. Gibaja, M. Eulàlia Subirà, Xavier Terradas, F. Javier Santos, Lidia Agulló, Isabel Gómez-Martínez, Florence Allièse, Javier Fernández-López de Pablo

**Affiliations:** 1 Archeology of Social Dynamics, Institución Milá y Fontanals, Consejo Superior de Investigaciones Científicas (IMF-CSIC), Barcelona, Spain; 2 Grup de Recerca Aplicada al Patrimoni Cultural (GRAPAC), Unitat d’Antropologia Biològica, Departament de Biologia Animal, de Biologia Vegetal i d’Ecologia, Universitat Autònoma de Barcelona, Bellaterra, Spain; 3 Centro Nacional de Aceleradores (Universidad de Sevilla, CSIC, Junta de Andalucía), Sevilla, Spain; 4 Unité Mixte de Recherche, 7041-Archéologies et Sciences de l’Antiquité (ArScAn), Université de Paris 1 Panthéon-Sorbonne, Nanterre, France; 5 Institut Català de Paleoecologia Humana i Evolució Social (IPHES), Tarragona, Spain; 6 Área de Prehistoria, Universitat Rovira i Virgili (URV), Tarragona, Spain; University of Toronto Mississauga, CANADA

## Abstract

Located on the Iberian Mediterranean coast, El Collado is an open-air site where a rescue excavation was conducted over two seasons in 1987 and 1988. The archaeological work excavated a surface area of 143m2 where 14 burials were discovered, providing skeletal remains from 15 individuals. We have obtained AMS dates for 10 of the 15 individuals by means of the direct dating of human bones. The ranges of the probability distribution of the calibrated dates suggest that the cemetery was used during a long period of time (781–1020 years at a probability of 95.4%). The new dates consequently set back the chrono-cultural attribution of the cemetery from the initial proposal of Late Mesolithic to an older date in the Early Mesolithic. Therefore, El Collado becomes the oldest known cemetery in the Iberian Peninsula, earlier than the numerous Mesolithic funerary contexts documented on the Atlantic façade such as the Portuguese shell-middens in the Muge and Sado Estuaries or the funerary sites on the northern Iberian coast.

## Introduction

In Europe, the recurrent use of specific spaces for a funerary use is attested with the last hunter-gatherer populations. These communities experienced significant changes in terms of diet diversification, patterns of residential mobility and demographic behaviour [1–10]. A cemetery is defined as a particular place, recognizable and recognized by a social group, where all or part of its community is buried over a period of time. This does not mean that the same place was not also used as a dwelling site. As E. Elder says: “cemeteries do not depend on a separation of the living and the dead” [9]. Indeed, in the Iberian Peninsula, both Mesolithic and Neolithic cemeteries are usually located in the same places as where domestic activities were carried out. Therefore, here we shall only consider funerary sites where at least two individuals are found in a primary position. Apart from certain exceptions, in the Iberian Peninsula, France or Italy, in the Upper Palaeolithic is usual to find isolated or disturbed burials, as well as bones with no anatomical connection [11–12]. This situation changes radically in the Mesolithic, when repeated burials took place in different kinds of funerary sites, in the open-air, caves and rock-shelters (see for instance [13–14]).

**Figure 1:**
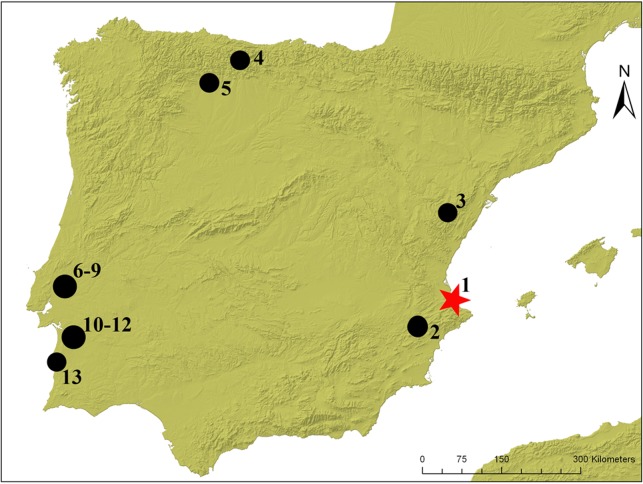
Location of the most important Mesolithic cemeteries with two or more individuals in a primary position. 1. El Collado, 2. Casa Corona, 3. Mas Nou, 4. Los Canes, 5. La Braña, 6–9. Muge Area (Cabeço da Amoreiras, Moita de Sebastião, Cabeço da Arruda, Cova de Onça), 10–12. Sado Area (Amoreiras, Arapouco, Cabeço do Pez), 13. Samouqueira.

In the Iberian Peninsula, three main regions are known with a rich funerary record associated with the last hunter-gatherer communities in the Mesolithic (Fig. 1):

- The Atlantic coast of Portugal. Large numbers of burials have been documented in the numerous shell-middens located on the banks of the estuaries of the Rivers Tagus and Sado, and on the south-west coast of Portugal. In the Muge area (mouth of the River Tagus), for example, shell-middens like those of Moita de Sebastião, Cabeço da Arruda and Cabeço da Amoreiras have surface areas of up to 3000m^2^ and a stratigraphic sequence nearly 5m thick. Over 300 Mesolithic individuals have been found in them, and dated to the period from 8500 to 7110 cal BP [8, 15–19]. The origin of the first Mesolithic cemeteries has been linked to changes in the settlement pattern as a consequence of the climatic changes caused by the 8.2 BP event. The groups systematically occupied the estuaries in order to intensify the exploitation of intertidal aquatic resources.
- In the north of the Iberian Peninsula (Cantabrian coast), no large cemeteries have been documented, possibly because the burials took place in small caves and rock-shelters. It is therefore usual to find a few graves, isolated burials or some dispersed bones. The most significant examples have been found at the cave sites of Los Canes, with three graves with the remains of five individuals, as two of the burials were double, and Los Azules, with one individual, both in Asturias [20–22], Aizpea, with one individual, in Navarre [23], the J3 shell-midden in Guipúzcoa, with one individual [24] and El Truchiro, with one individual, in Cantabria [25]. In the north of Spain, but inland, the burials of two individuals have recently been discovered in the Cave of La Braña, León [26–27].
- Finally in eastern Spain, burials are known at the rock-shelter of Mas Nou (Castellón) with 7 individuals, the open-air site of Casa Corona (Alicante) with two burials, la Peña del Comptador (Alicante) also with two partial burials at the base of a long wall and especially the open-air site of El Collado (Valencia) with a necropolis with 14 graves, one of which contained remains of two individuals [28–32].

The aim of this paper is to present the results of the C14 (AMS) dates obtained for 10 of the 15 individuals at the site of El Collado, the largest Mesolithic funerary site in Spain. Although largely unknown outside Spain, the site is important because of the number of individuals buried, most of them in individual graves and in primary position.

The new AMS radiocarbon dates conducted on bone collagen samples of human remains have defined the different chronological phases of funerary activity and consequently the chrono-cultural attribution of the burials had to be revised. The comparison of the results with the radiocarbon record of Mesolithic burials in Portugal and northern Spain reveals the older age of the cemeteries in the Iberian Mediterranean area.

## The Mesolithic Site of El Collado

The site of El Collado is located on the southern side of the Gulf of Valencia, at about 100m above sea level and 3km from the modern shoreline. It is an open-air settlement on a hillside, in the shelter of an outcrop of large limestone blocks. The available data on the evolution of this sector of the Mediterranean coast indicate that a marine transgression at about 8300±170 BP reached its maximum height in the Flandrian, in 6130±100 BP [33]. These changes in the shoreline led to the development of several areas of wetlands, in the form of coastal lagoons and marshes in the immediate surroundings of the site. Its location would have allowed the human communities easy access to a range of terrestrial and marine ecosystems.

Discovered in the early twentieth century [34], the site has only seen two seasons of rescue excavations in 1987 and 1988, when the burials studied in this paper were found. This fieldwork, directed by José Aparicio, excavated a surface area of 143m^2^. A total of 14 burials in a shell-midden type of archaeological deposit were documented, together with numerous lithic artifacts, faunal and malacological remains. No grave goods have ever been cited [28].

**Figure 2:**
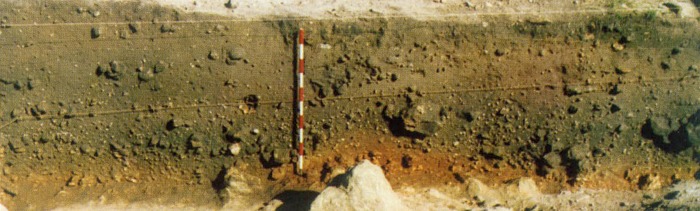
Stratigraphy at El Collado (Section B-C). Photograph published in Aparicio 2008 [28], page 112.

The stratigraphic sequence at El Collado exhibits a variable depth, between 1 and 1.5m thick. Despite the lack of sedimentological studies to explain the deposit formation process, the geomorphologic characteristics of the surroundings and the photographs of the stratigraphic sections suggest that it formed by colluvial sedimentary deposition and anthropic sedimentation. The stratigraphic sequence consists of four levels whose characteristics are summarized in Table 1 and Fig. 2 [28]. Level I corresponds to the Late Mesolithic, with a lithic assemblage characterized by trapezes, which in the regional sequence is dated to between 8600–8000 cal BP [35]. Level II, which is the thickest level, is dated in the Early Mesolithic, also known as the “Notches and Denticulates Mesolithic”, which is dated regionally to 10,600–8600 cal BP [36–37]. Levels III and IV represent the lower part of the sequence. Level III is partially disturbed and is the least thick, with a similar assemblage to that in Level II. Finally, Level IV, at the base of the sequence, has yielded a small number of backed bladelets suggesting an Epipalaeolithic attribution, according to the regional sequence.

**Table 1:** Synthetic description of the stratigraphic sequence at El Collado, based on the interpretation of the published lithic assemblages [28] and recent unpublished studies of Squares FIII, FIV, FV and GIII.

**Level**	**Description**	**Thickness**	**Cultural Affiliation**
I	Blackish-gray (disturbed) sediment	10–50 cm	Late Mesolithic (trapezes)
II	Dark brown sediment	60–80 cm	Early Mesolithic (Notches and Denticulates)
III	Basal brown-reddish sediment	10–20 cm	Early Mesolithic (Notches and Denticulates)
IV	Reddish sediment	20–30 cm	Epipaleolithic

**Figure 3:**
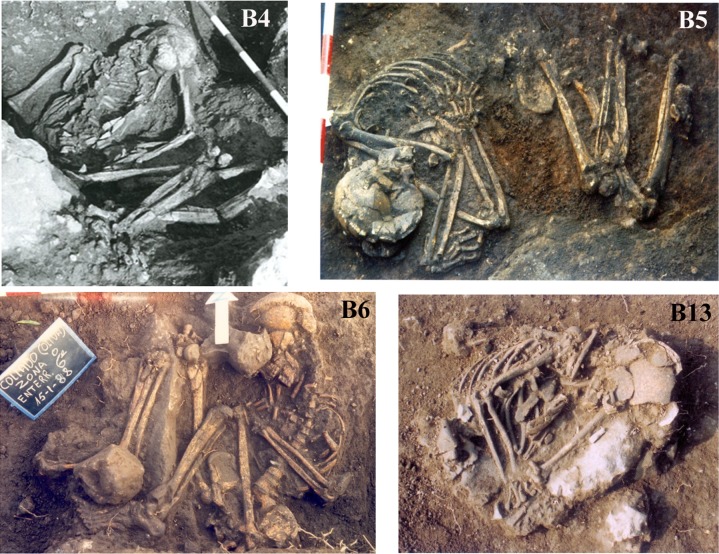
Photographs of Graves 4, 5, 6 and 13 in El Collado cemetery.

**Figure 4:**
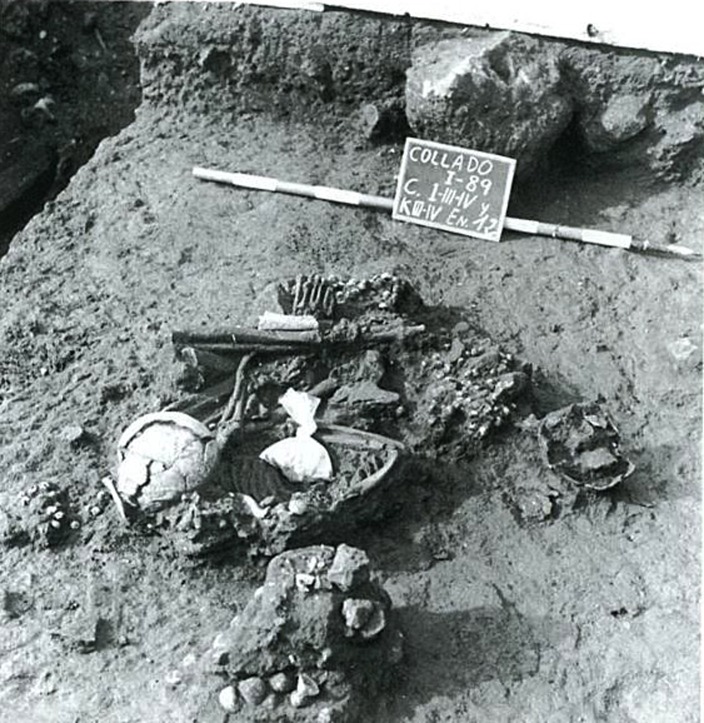
The individual found in a primary position in Grave 12 was possibly bound up when buried (Photograph in Aparicio 2008 [28]: 135).

The funerary record at El Collado consists of 14 graves, of which 13 are primary individual burials and the other (Fig. 3), Grave 12, held an individual in a primary position and an isolated skull in a secondary position (Fig. 4).

There is very little information about the position of the burials in the stratigraphic sequence due to the excavation method and the recording system which was developed. The stratigraphic interface of each burial pit was not either recorded during the excavation process. Thus, the correlation between the burials and the stratigraphic sequence relies on the written descriptions of the fieldwork director. According to Aparicio [28], the burials recovered during the first fieldwork season (burials 1–9) were associated with Level II, even though the pit burials partially cut Level III. In contrast, burial 10 was completely buried in Level II. Consequently, burial 10 stratigraphically postdates burials 1–9, despite the fact that the relative chronological position amongst burials 1 to 9 is impossible to elucidate with the published information. On the other hand, burials 11 to 14, recovered during the second fieldwork season, were also associated with Level II. However, the position of the putative burials inside Level II was different, according to a schematic stratigraphic section [28], as they were related to two different layers within Level II: burial 12 to Level II-layer 1, and burials 10, 11, 13 and 14 to Level II-layer 4. According to this information, all the funerary activity at the site took place during the formation of Level II, suggesting an a priori Early Mesolithic (of notches and denticulates affiliation) cultural attribution.

The analysis of the photographs and plans from the 1987 and 1988 excavations has shown that at least nine of these individuals were buried in a flexed or hyper-flexed position (Fig. 4). The position of the skeletal remains and the movements documented suggest not only that the bodies decomposed in a filled space but that they were buried in some kind of shroud or sack or were tied [38–39].

Four of the individuals were females and seven were male. The morphological characteristics of the individual in Grave 8 and the isolated skull in Grave 12 suggest that they also were males. The sex of the two youngest individuals, in Graves 9 and 10, cannot be determined. One of them died around the time of birth, and the other during adolescence (Table 2).

**Table 2:** Sex and age at death of the individuals.

**Burial**	**Sex**	**Age**	**Preservation**	**%C**	**δ^13^C**	**δ^15^N**	**C/N**
1	♀	adult	26%	23,7	-19,5	10,2	3,4
2	♀	adult	32,5%	24,9	-19,1	8,9	3,3
3	♂	20–45 y	33,5%	15,9	-17,6	10,2	3,2
4	♂	30–40 y	73,5%	24,1	-17,6	12,8	3,4
5	♀	20–25 y	19,5%	21,7	-18,2	10,6	3,3
6	♂	20–25 y	75%	27	-18,2	10,9	3,3
7	♀	35–40 y	63%	29,9	-17,9	8,9	3,4
8	♂?	15–18 y	5%	-	-	-	-
9	-	young	-	-	-	-	-
10	-	9 months	21,4%	-	-	-	-
11	♂	18–22 y	60%	-	-	-	-
12	♂	40–45 y	47,5%	37	-19	9,5	3,5
12, 2nd skull	♂?	young	-	-	-	-	-
13	♂	18–22 y	41,5%	19,6	-18,1	10,4	3,3
14	♂	>40 y	50%	-	-	-	-

The percentage conserved of each individual and the δ^13^C and δ^15^N values obtained by the isotope analysis are also given.

Most of the individuals were between 20 and 30 years of age, although four of them were older, in some cases over 40 years old: the male individuals in Graves 4, 12 and 14, and the female in Grave 7. All the sub-adults were males; one between 15 and 18 years of age, two 18–22 years of age and a further two, the individual in Grave 9 and the skull in a secondary position in Grave 12, display no criteria to define their age at death. In short, the adult individuals in the population are represented above all, and most are male individuals, with only one newborn. The population displays some morphological traits, such as naso-alveolar prognathism, considered archaic and suggesting a local population from a previous period [40–41].

A preliminary trace element study proposed that the population’s diet was rich in products with a marine origin [42]. A later study of δ^13^C and δ^15^N stable isotopes in bone collagen samples taken from nine individuals indicated a mixed diet with differing amounts of marine proteins, which reached 25% in two of the individuals. This proportion was smaller, or the diet was primarily terrestrial in the case of the others [43] (Table 2).

Finally, as the teeth were badly worn, a study of dental micro-striations examined the possible use of the teeth for a non-alimentary purpose. This study of the dental alterations showed that the population at El Collado used their teeth as a kind of tool [44]. The orientation and length of the striations in the pre-molars and canine teeth showed they were used in the treatment of plant fibres. This work was identified in the whole population, and thus the individuals in the group habitually used their teeth in para-masticatory tasks throughout their life [45].

It is therefore a population with an ancient substrate, exhibiting continuity with the previous populations in their daily activities. However, there is a change in their burial ritual.

## Materials and Methods

### 1. Data acquisition and sample selection protocols

The Accelerator Mass Spectrometry radiocarbon dates were performed at the Centro Nacional de Aceleradores (CNA) in Seville, Spain. Samples from human bones for isotopic studies and dating were taken from the Valencia Prehistory Museum with the permission of authorities in charge, according to the Spanish legislation for historical Heritage.

As they are single burials in primary position, all the human remains were grouped and identified by their grave number during excavation, without specific inventory numbers being given to each bone. The samples were selected from compact bone with no diagenetic alterations nor with consolidants or adhesive substances. Wherever possible, the lower limb was chosen because of the greater bone density and consequent greater possibility of conserving collagen. According to these criteria, ten individuals were selected for radiocarbon dating. Data about the grave, the bone selected and its weight are listed in Table 3.

**Table 3:** Samples dated, with the grave number, bone type and sample weight.

**Burial**	**Weight**	**Bone**
Burial 1	29,8 g	Femur
Burial 3	33,6 g	Humerus
Burial 4	81,3 g	Tibia
Burial 5	15,3 g	Tibia
Burial 6	18,0 g	Tibia
Burial 7	30,3 g	Tibia
Burial 9	18,6 g	Diaphysis and Skull
Burial 11	18,7 g	Femur
Burial 12	15,0 g	Diaphysis
Burial 13	45,3 g	Tibia

### 2. Pretreatment methods

Bone samples were first pretreated in the laboratory (Unitat d’Antropologia Biològica, Universitat Autònoma de Barcelona) in order to clean them of soil and other adhered substances using water and mechanical elements. Once cleaned, the following steps were taken to extract and purify the collagen in the bone, since this fraction is thought to give the most reliable results. The mineral part of the bone is more subject to contamination due to interaction with the surrounding environment. The whole process includes several steps detailed in other publications [46–49], and basically consists of the demineralization of the bone and subsequent purification steps to extract the collagen. One of these purification steps is the so-called ultrafiltration of the collagen, which eliminates the low weight protein chains from collagen, which in some cases may be contaminated. However this procedure reduces the collagen yield [50] and there is still a live discussion about the best procedures and convenience of the ultrafiltration procedures [47, 51–54].

In this case a preliminary test was made on three bones in order to check whether ultrafiltration introduced a difference in age, since low collagen yields were expected. Subsamples of these bones were prepared following three different methods and AMS dated. The first method consisted of a simple modification of the one proposed by Longin [55]. After demineralization of the bone, gelatine was neutralized and a solution of NaOH 0.1M was added at room temperature for 15 minutes, in order to remove potential humic contaminants. Gelatine was neutralized and solubilised in HCl at pH = 3 overnight at 80ºC. Remnants were eliminated by centrifugation and the solution was dried to obtain the final collagen.

The second method is described in García-Guixe *et al.* 2006 [43], and includes ultrafiltration, but no basic bath. Finally, the third method is the same as the second but includes the basic bath before solubilisation.

The preliminary tests using these pre-treatment methods showed no significant differences in the radiocarbon ages. Given the confirmed low collagen yields, we decided to use the most conservative method for the samples.

Each sample was combusted in an elemental analyzer where were the carbon dioxide was separated and purified to be transferred to an AGE graphitization system designed at the AMS unit of the Institute of Particle Physics at ETH Zürich [56]. Here, carbon dioxide is mixed in a reactor with hydrogen in the presence of iron as a catalyst. The reaction takes place at high temperature and graphite is deposited over the catalyst. Water is produced and is trapped by a cold finger. The graphite produced is pressed in an aluminum piece and is ready to be measured.

Samples for radiocarbon dating can be measured at two different facilities at CNA. SARA (Spanish Accelerator for Radionuclide Analysis) [57] has been in use since 2006, while Micadas (Mini Carbon Dating System) [56] was installed in 2012, and is currently used as the default facility for radiocarbon dating. Both systems follow the same AMS principles. Graphite samples were sputtered by a caesium beam in order to obtain a negative ion beam from the sample, and several kinematic filters were used in order to eliminate undesired components of the beam. In order to achieve the necessary sensitivity, molecules were broken in the stripper tube at the high voltage terminal.

Stable isotopes ratios were measured at the high energy side using Faraday cups. Thus, an isotopic ratio was obtained for each sample, which can be compared to standard samples of known ratio in order to normalize, and background samples to determine the maximum sensitivity. Typical background values correspond to *ca*. 45,000–50,000 years, and modern samples can be measured to about 2–3‰ level.

Results from Micadas were analyzed with a particular software designed for Micadas called BATS [58] to obtain the corresponding radiocarbon age (Age BP), following Stuiver and Polach 1977 [59]. Radiocarbon dates were calibrated and plotted using Oxcal 4.2 [60] software and a mixed marine and atmospheric calibration curve [61] on the basis of a local marine reservoir value and the percentage of marine diet calculated for each individual [43].

In order to take into account possible regional differences in the marine curve, the ∆R parameter is used [62]. Although there are no published ∆R values for this specific area of the Mediterranean, there are data for some close locations [63–64]. An average value of ∆R = 94±61 was calculated using the Marine Radiocarbon database at www.calib.qub.ac.uk/marine [65] and used as an estimation for the location of our study. This value is included in the calibration data.

## Results

### 1. Radiocarbon results and Bayesian phase modeling

**Figure 5:**
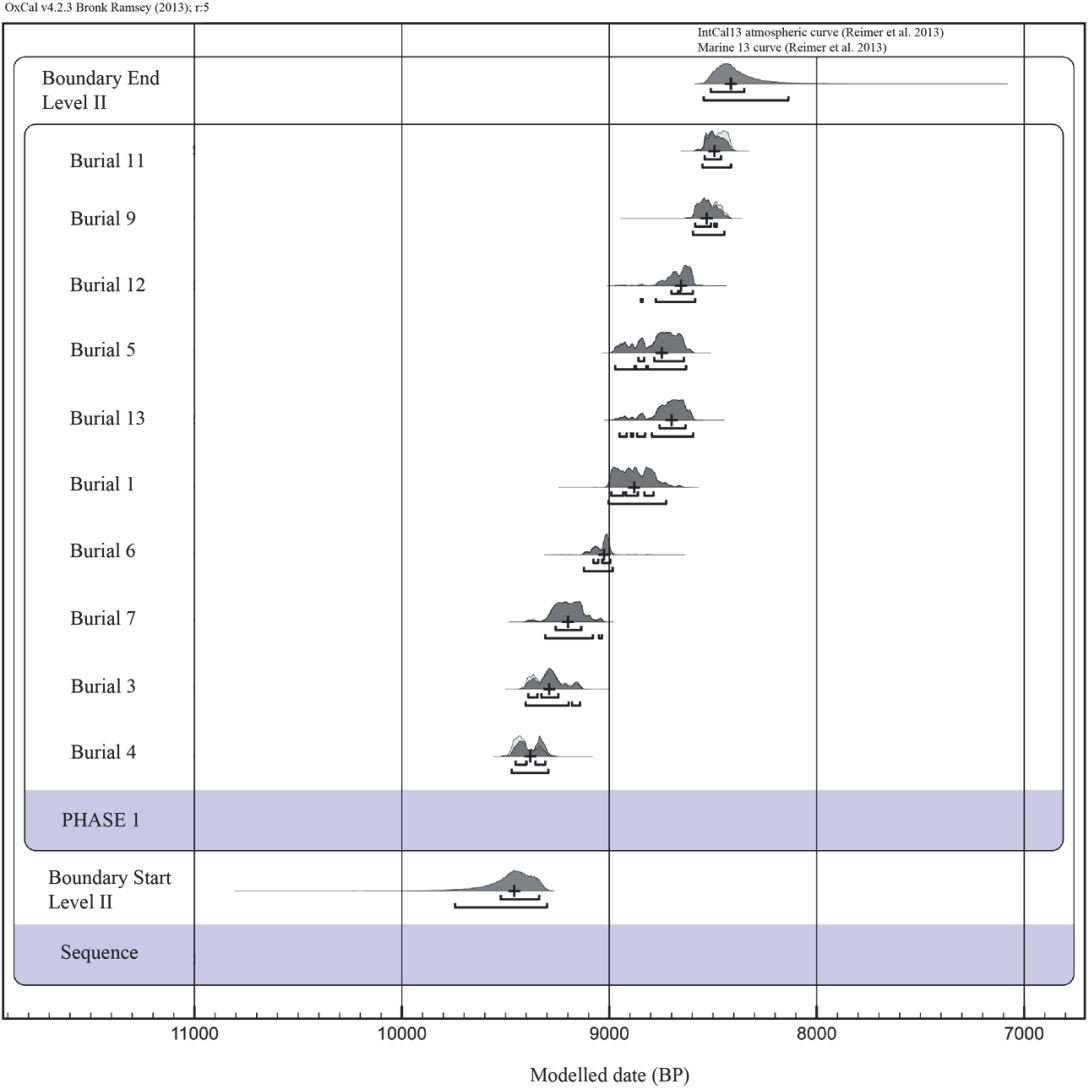
Bayesian phasing model plot of the El Collado level II sequence based on radiocarbon determinations of human skeletons (see Table 5). Light gray color represents the prior distributions, the dark gray the posterior distributions constrained to the phase. The cross represents the median of the posterior distribution ranges. The brackets indicate the Agreement index of each sample in the phase model.

The radiocarbon results are listed in Table 4, together with the percentage of marine diet inferred for each sample. For Burials 9 and 11, palaeodietary data were not available and, consequently, the percentage of marine diet could not be calculated directly. Oxcal 4.2 allows samples with unknown dietary values to be calibrated using a mixed marine-terrestrial calibration curve and stochastically simulating different percentages of marine diets. However, using such a procedure, the 2σ calibration ranges obtained for individuals 9 and 11 were significantly broader than the remaining samples (e.g. Burial 11: 8582–8046 cal BP; Burial 9: 8640–8073 cal BP). These broad calibration ranges are, in fact, an artefact of the uncertainty regarding the unknown percentage of marine diet associated with both samples. In order to narrow the chronological ranges of Burials 11 and 9, and to constrain them to a more realistic dietary estimation, we decided to compute for both samples the mean percentage of marine diet reported for all the individuals analysed at El Collado (mean = 13.75). Therefore, using the mean values, the 2σ unmodeled calibration ranges obtained were significantly narrower (e.g. Burial 11: 8543–8408 cal BP; Burial 9: 8591–8435 cal BP). To test the internal consistency and stratigraphically constrain the chronological ranges produced by the radiocarbon calibrations, we used the Oxcal 4.2. phase model. Using this kind of analysis, we assume that our radiocarbon data set consists of stratigraphically unordered graves buried during the formation of Level II. In addition, using a phase model we assume the absence of direct stratigraphic superimposition between the dated burials. Such an assumption also considers the potential stratigraphic disturbance produced by the repeated excavation of pits over a reduced area, and fits with the fieldwork observations briefly reported by the excavator. The results of the phase model analysis are reported in Table 5, where both the unmodeled and phase modeled chronological ranges at the 95.4% confidence level and the start and end phase boundaries are detailed. The burial calibration ranges are represented graphically in Fig. 5 as modeled probability distributions. The phase model produced acceptable agreement indexes (A_model_ = 99.6; A_overall_ = 99.8), well above the critical value of Ac = 60. The 2σ modeled phase boundaries for Level II were 9744–9300 cal BP for the boundary start and 8545–8137 cal BP for the boundary end. Both boundaries probabilistically indicate the chronological limits for the formation of Level II, within which the burial activity took place. Such chronological limits reinforce the cultural attribution of Level II as Early Mesolithic, which agrees with the cultural attribution of the lithic assemblage, even though the end boundary partially overlaps the chronological range of the Late Mesolithic trapeze phase in the central Mediterranean region of Spain [35] built on non-human short-lived samples (8580–8040 cal BP).

**Table 4:** AMS radiocarbon dates on bone collagen of the human burials from El Collado.

**Lab. Code**	**Burial**	**Age BP**	**δ^13^C (‰)^1^**	**δ15N** **(‰)**	**Marine diet (%)**	**δ^13^C** **(‰)^2^**	**Yield**	**C/N**	**2 σ cal BP Age**
1619.1.1	1	8067±34	-19,50	10.2	0	-20,5	0,21	3,4	9090–8780
1620.1.1	3	8388±36	-17,60	10.2	25	-21,9	1,69	3,2	9401–9134
1621.1.1	4	8491±37	-17,60	12.8	25	-22,4	1,09	3,4	9475–9300
1622.1.1	5	7992±34	-18,20	10.6	17	-17,8	0,14	3,3	8970–8606
1623.1.1	6	8166±35	-18,20	10.9	17	-20,9	2,25	3,3	9129–8811
1624.1.1	7	8319±35	-17,90	8.9	21	-18,9	0,45	3,4	9298–9033
1625.1.1	9	7801±38	nd	nd	13.5	-26,6	0,12	nd	8591–8435
1626.1.1	11	7742±35	nd	nd	13.5	-22,8	0,14	nd	8543–8408
1627.1.1	12	7900±32	-19,00	9.5	7	-14,7	0,33	3,5	8844–8582
1628.1.1	13	7976±33	-18,10	10.4	19	-17,2	0,57	3,3	8947–8592

**Table 5:** Bayesian phasing model of the El Collado Level II sequence based on radiocarbon determinations of human skeletons.

**Amodel 99.6** **Aoverall 99.8”**	**Unmodelled (BP)**						**Modelled (BP)**					
	**from**	**to**	**%**	**from**	**to**	**%**	**from**	**to**	**%**	**from**	**to**	**%**
Boundary End level II							8510	8349	68.2	8545	8137	95,4
R_Date Burial 11	8516	8424	68.2	8545	8409	95,4	8540	8462	68.2	8551	8413	95,4
R_Date Burial 9	8583	8477	68.2	8594	8433	95,4	8586	8482	68.2	8597	8445	95,4
R_Date Burial 12	8701	8598	68.2	8850	8585	95,4	8701	8598	68.2	8847	8586	95,4
R_Date Burial 5	8861	8640	68.2	8972	8630	95,4	8860	8640	68.2	8972	8630	95,4
R_Date Burial 13	8764	8631	68.2	8953	8595	95,3	8758	8632	68.2	8950	8596	95,4
R_Date Burial 1	8991	8789	68.2	9006	8726	95,4	8991	8788	68.2	9004	8726	95,4
R_Date Burial 6	9080	8995	68.2	9128	8978	95,4	9077	8995	68.2	9123	8984	95,4
R_Date Burial 7	9261	9134	68.2	9370	9033	95,4	9258	9135	68.2	9309	9035	95,4
R_Date Burial 3	9397	9249	68.2	9405	9140	95,4	9390	9246	68.2	9403	9141	95,4
R_Date Burial 4	9472	9327	68.2	9484	9304	95,4	9452	9309	68.2	9471	9295	95,4
Boundary Start level II							9523	9338	68.2	9744	9300	95,4

All radiocarbon dates have been calibrated using the mixed INTCAL 13 and Marine13 calibration curve considering a ΔR of 94±61 for the Western Mediterranean. The percentage of marine diet has been calculated from García-Guixé *et al.* [43] according to δ^13^C (‰)^1^. δ^13^C (‰)^2^ is the value obtained from AMS measurement, and is related to the graphite and not directly to collagen.

Once established the start and end boundary of Level II, the Span function of Oxcal was used to determine the chronological span of the dated burials contained in Level II, in other words, the chronological span in which the funerary activity took place based on the modeled calibration ranges of each dated burial. The span result ranges between 781 and 1,020 years (at a probability of 95.4%).

### 2. Revision of previous (conventional) radiocarbon dates

The *X^2^* test, carried out with the R_Combine function of Oxcal (vers. 4.2) shows that the analyzed series is made up of statistically different dates (df = 10 T = 515.652 (5% 18.3).

Thus, Burial 4 is the oldest in the series (9475–9300 cal BP), while Burial 11 gave the most recent date (8543–8408 cal BP). Taking the upper limit of the oldest date and the lower limit of the youngest date, at a probability of 95.5%, it may be concluded that the individuals in the necropolis were buried at different times within a period of nearly 1100 years. The new AMS determinations have allowed a new assessment of the four conventional radiocarbon dates. These dates correspond to the individuals in Graves 4, 6 and 13 [28]. If these burials are examined individually (Fig. 5 and Table 4) it may be seen that the individual in Grave 4 provided a conventional radiocarbon date about 200 years older than the AMS determination. However, the calibrations of the dates obtained for this individual in each laboratory give age ranges that overlap partially. The same can be said for the individual in Grave 6, with the difference that this time the conventional determination is more recent than the AMS one (8166±35 BP C14/AMS and 8080±60 C14 Conventional).

However, the largest age differences are undoubtedly those of the determinations obtained for Burial 13. Two samples taken from this individual were dated by the conventional method in the early 1990s, and they obtained dates characterized by a high standard deviation (7640±120 BP and 7570±180 BP). After calibration, the age ranges are too large (nearly 1000 years). The new date obtained by the AMS method sets back the date of this burial 300 years and reduces the age range considerably after calibration at 2σ. The date of this individual means that the interpretation of El Collado as a Late Mesolithic cemetery, as concluded in the past when only the dates for Burial 13 were available [35], needs to be reconsidered.

The new dates therefore improve the chronological resolution of the different individuals noticeably and consequently set back the chrono-cultural attribution of the cemetery from the initial proposal of Late Mesolithic to an older date in the Early Mesolithic.

### 3. Phases of funerary activity at El Collado cemetery

The modeled probability distributions of the calibrated radiocarbon dates indicate the cemetery was in use for a long period of time. The visual inspection of the chronological ranges of the calibrated dates suggests that the cemetery was used in several phases.

- The oldest phase is represented by Burials 4, 3 and 7, which are dated to a period lasting approximately 200 years, between 9475 and 9033 cal BP. The chronology of Burial 4 is slightly older than Burials 3 and 7. However, age ranges at probabilities of 95.4% obtained by the calibrations of Burials 4 and 3 partially overlap.
- A second group of dates is represented by Burials 1, 5, 13 and 12, whose calibrated dates overlap partially. They indicate the time of the maximum use of the site as a cemetery, from 9090 to 8582 cal BP.
- Between these two phases, Burial 6 occupies an intermediate position (9129–8811 cal BP), which only attests the continuous use of the cemetery.
- Finally, the determinations of Individuals 9 and 11 produced statistically similar results, representing the last phase of the use of the cemetery, between about 8591 and 8408 cal BP.

**Figure 6:**
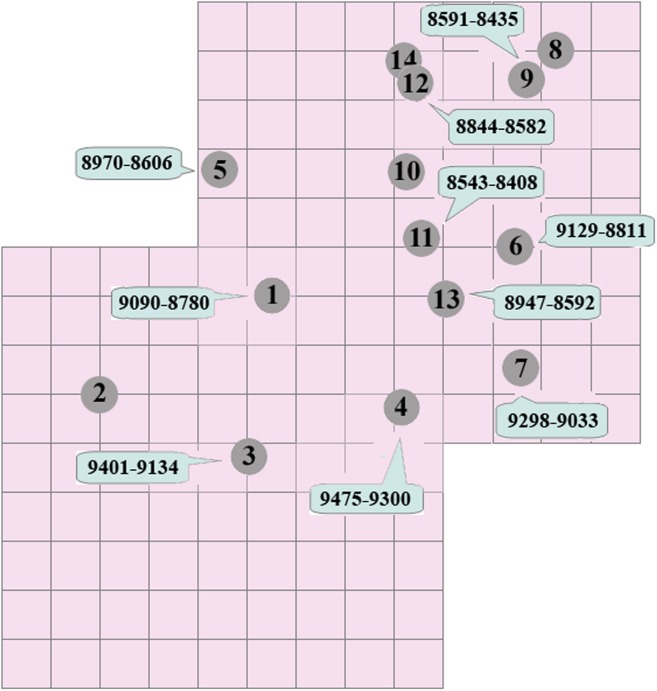
Plan with the position of the graves at El Collado and the corrected BP dates (except Individuals 9 and 13, for which no isotope data is available. See Table 4).

Additionally, by comparing the chronological data with the positions of the graves, it can be seen that these are organized to a certain extent from south to north (Fig. 6). Thus, whereas the oldest burials are in the south, the most recent tend to be in the northernmost part of the site. Together with the general absence of intersections, where one grave cuts another (the only exceptions are Graves 12 and 14), this suggests that the Mesolithic community was somehow aware of the layout of the cemetery. Given the small space in which the tombs are located, a plausible explanation is that the graves were marked in some way, avoiding the total or partial destruction of previous burials.

## Discussion and Interregional Comparisons

The body of AMS radiocarbon dates presented in this work sheds new light on the chronology of funerary practices and the emergence of cemeteries in south-western Europe. First, with 10 radiocarbon determinations from 10 different individuals, El Collado is the Iberian Mesolithic cemetery with largest number of dated burials. As previously discussed in the result section, El Collado witnessed prolonged funerary activity, which implies the persistent and repeated use of this cemetery, spanning almost a millennium (781–1,020 years at 95.4% probability according to the Bayesian phase modeling). Such an extended use is unknown at other Iberian Mesolithic cemeteries, where the funerary activity lasted just a few generations [19].

Besides the extended funerary use of El Collado during a millennium aproximately, the new radiocarbon dates uncover a new interesting macro-regional phenomenon: the first burial phase at El Collado -represented by Burials 4, 3 and 7- not only does it constitute the oldest Mesolithic cemetery known in the Iberian Peninsula, but it also suggests that the emergence of cemeteries between the Mediterranean and the Atlantic regions of Iberia followed a very different chronological pattern.

**Figure 7:**
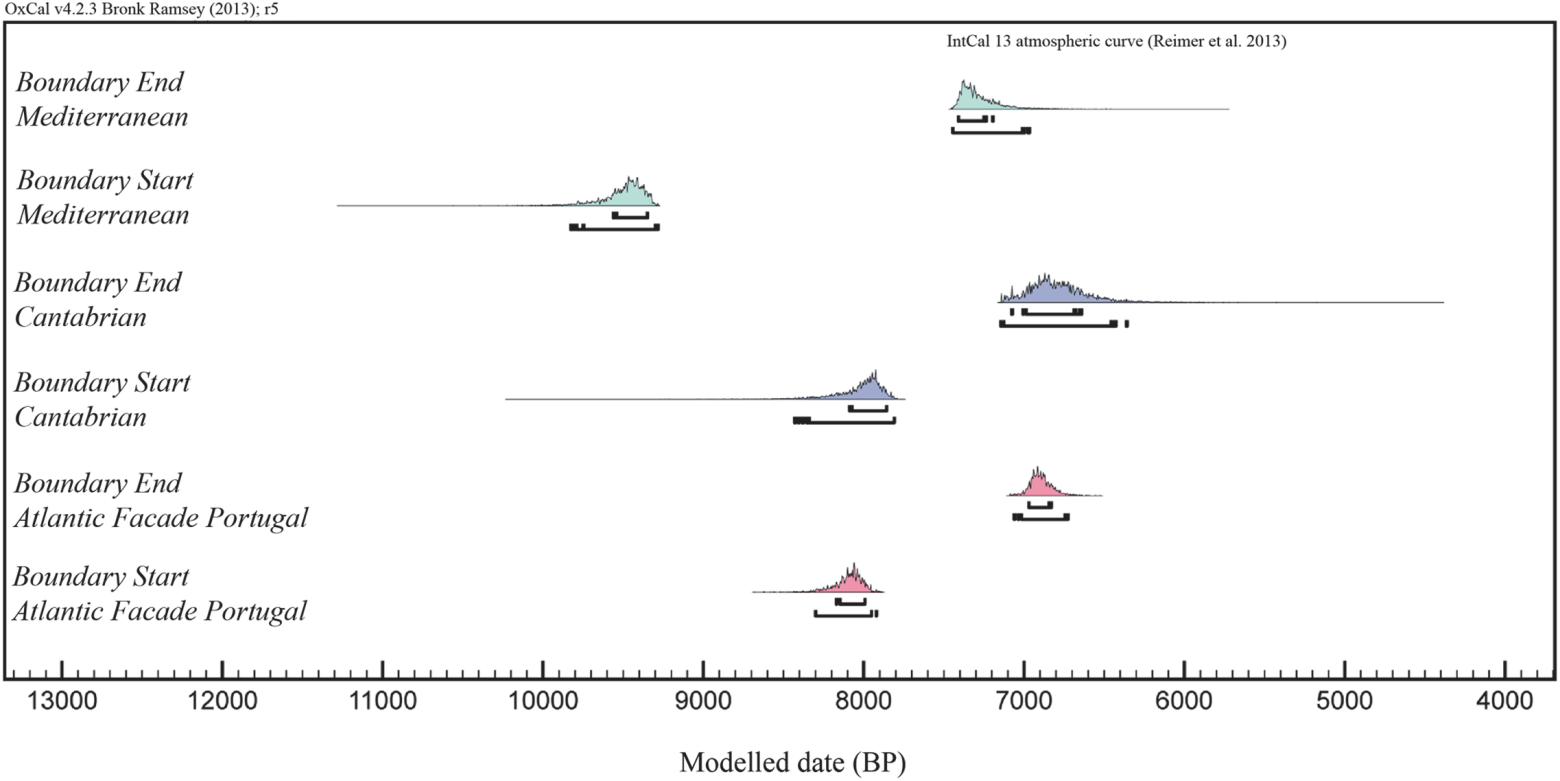
Plot of the Oxcal three phase overlapping model displaying the inter-regional comparison of chronological limits of Mesolithic cemeteries in Iberian Mediterranean, Cantabrian and Portuguese regional units. Model built on individual radiocarbon samples of Mesolithic skeletons found at cemeteries (see SM1 for details and Table 6).

To support this hypothesis, we have conducted an interregional analysis of the radiocarbon record of Mesolithic skeletons from all the Iberian cemeteries. Our analysis is constrained to those sites with more than one primary burial. Therefore, individual burials occasionally found at some rock-shelters, as well as dispersed human remains documented in archaeological layers, have been omitted. The audited data set consists of 47 radiometrically-dated skeletons from 13 different cemeteries. The supporting information for each dated skeleton is provided in S1 Table. Only dates with standard deviations ≤±100 years obtained for human bone samples are given. In this way, it is certain the dates being compared were obtained for the individuals and not for grave goods or other objects associated with them, such as charcoal, shells, fauna, etc [66–67]. All the radiocarbon dates have been calibrated as in El Collado data set, considering the percentage of marine diet and using a mixed marine-terrestrial calibration curve with different local ∆R values. In addition, in order to compare the initial chronology of cemeteries regionally, we have created a three phase overlapping model assembling the radiocarbon dates by region (Mediterranean = 16, Portugal = 25, Cantabrian = 6). The results are summarized in Table 6, with start/end boundaries that can be compared between regions, and graphically displayed in Fig. 7.

**Table 6:** Oxcal three phase overlapping model with the 95.4% distribution chronological ranges of the boundary start and end in the Iberian Mediterranean, Cantabrian and Portugal regional units.

	**Modelled (BP) 2 σ**		**Median**
Mediterranean Boundary End	7442	6958	7312
Mediterranean Boundary Start	9801	9283	9463
Cantabrian Boundary End	7128	6385	6808
Cantabrian Boundary Start	8367	7812	7983
Portugal Boundary End	7039	6738	6902
Portugal Boundary Start	8292	7893	8074

Model built on individual radiocarbon samples of Mesolithic skeletons found at the Iberian cemeteries (see SM1 for details and Fig. 7 for the visual display of the distribution ranges).

The most significant difference between the Mediterranean region and the Cantabrian region and Portugal is seen in the appearance of cemeteries during the Early Mesolithic. In addition, by assessing the data from the three areas in the Iberian Peninsula the following chronological patterns can be identified.

- In the Mediterranean region, the dates from El Collado show that cemeteries appeared about 9475–9300 cal BP. This type of funerary practice will continue in other nearby sites, such as Casa Corona [31] and Cingle del Mas Nou [29]. Unlike El Collado cemetery, which was in use for some 1,100 years, according to the dates obtained, at Casa Corona and Cingle del Mas Nou, their time of use is much shorter (8007–7583 cal BP). In addition, Cingle del Mas Nou is different from the other Mesolithic burial sites in the Iberian Peninsula as the remains of seven individuals (whole and incomplete) were deposited in a single structure.
- On the Atlantic facade of Portugal, the first evidence of cemeteries in the Muge Estuary date to 8409–8030 cal BP (at Cabeço de Arruda, for example). These are associated with large shell-middens occasionally over 5m thick, in use over a long period of time [8, 15]. In the Sado Estuary the dates are slightly more recent than at Muge, beginning about 8200 cal BP (e.g. at Amoreiras). It is clear that from 8160–7970 cal BP, the Mesolithic groups systematically buried all or some of their dead in cemeteries.
- Finally, the dates for funerary sites in northern Iberia (Cantabrian coast) with two or more individuals indicate that the first grouped Mesolithic burials were a little more recent (between 7981 and 6636 cal BP). In any case, it should be stressed that, unlike in the other two areas, at most sites only a single individual has been documented or much smaller groups, as at Los Canes and La Braña.

This paper has presented the new dates obtained for several of the burials at the site of El Collado and compared the results with those published for other Mesolithic sites in the Iberian Peninsula with primary burials. These have been documented in many different kinds of sites, from the large shell-middens on Portuguese estuaries, to smaller shell-middens in other parts of Iberia, caves in northern Spain and open-air sites.

The comparison of all the dates has shown that the burials at El Collado took place before those found at sites on the Atlantic and Cantabrian seaboards.

Further reflections on the significance of the establishment of cemeteries by Mesolithic hunter-gatherer communities go beyond the objectives of this paper. However, the repeated use of a place to bury the dead first occurred in this period and had a chronological continuity. As in the Mesolithic, during the Neolithic, farming communities also buried their dead in the same places where they lived. This is shown by the fact that funerary and domestic structures are documented in the same locations, as has been found at several sites in the Iberian Peninsula [13].

In conclusion, the results of the present study break with the conception that the first cemeteries in the Iberian Peninsula were created by the Mesolithic communities settled on the Atlantic coast of Portugal about 8400–8200 cal BP. By obtaining a series of dates from the cemetery at El Collado, it has been possible to determine not only the true age of this cemetery but also how the burial space was organised by hunter-gatherer groups living near the Mediterranean shores of the Iberian Peninsula.

A great deal of work remains to be done, and many doubts need to be solved regarding the site of El Collado; however this paper represents the big step forwards that has been taken and which will guide the future actions that are being planned.

## Supporting Information

S1 Table Radiocarbon dates of human skeletons found at the Iberian Mesolithic cemeteries from the Mediterranean, Atlantic and Portugal regional units. (DOC)

S1 Text Inter-regional comparisons of Iberian radiocarbon dates obtained from human bones. (DOC)
