# Supplementary material for: The Emergence of Mesolithic Cemeteries in SW Europe: Insights from the El Collado (Oliva, Valencia, Spain) Radiocarbon Record
Source: PLoS One. 2015 Jan 28;10(1):e0115505. doi: 10.1371/journal.pone.0115505 (PMC4309619; doi:10.1371/journal.pone.0115505)
Supplement: S1 Table — (DOC) [file pone.0115505.s001.doc]

| **Site** | **Individual** | **Lab Code** | **δ13C** | **δ15N** | **% Marine** | **ΔR** | **BP Age** | **SD** | **2σ cal BP age** | **Reference** |
| --- | --- | --- | --- | --- | --- | --- | --- | --- | --- | --- |
| **MEDITERRANEAN REGION** | | | | | | | | | | |
| **El Collado** | Burial 1 | CNA-1619.1.1 | -19.5 | 10.2 | 0 | 94±61 | 8067 | 34 | 9090-8780 | This study |
| **El Collado** | Burial 3 | CNA-1620.1.1 | -17.6 | 10.2 | 25 | 94±61 | 8388 | 36 | 9401-9134 | This study |
| **El Collado** | Burial 4 | CNA-1621.1.1 | -17.6 | 12.8 | 25 | 94±61 | 8491 | 37 | 9475-9300 | This study |
| **El Collado** | Burial 5 | CNA-1622.1.1 | -18.2 | 10.6 | 17 | 94±61 | 7992 | 34 | 8970-8606 | This study |
| **El Collado** | Burial 6 | CNA-1623.1.1 | -18.2 | 10.9 | 17 | 94±61 | 8166 | 35 | 9129-8811 | This study |
| **El Collado** | Burial 7 | CNA-1624.1.1 | -17.9 | 8.9 | 21 | 94±61 | 8319 | 35 | 9298-9033 | This study |
| **El Collado** | Burial 9 | CNA-1625.1.1 | nd | nd | 13.5 | 94±61 | 7801 | 38 | 8591-8435 | This study |
| **El Collado** | Burial 11 | CNA-1626.1.1 | nd | nd | 13.5 | 94±61 | 7742 | 35 | 8543-8408 | This study |
| **El Collado** | Burial 12 | CNA-1627.1.1 | -19 | 9.5 | 7 | 94±61 | 7900 | 32 | 8844-8582 | This study |
| **El Collado** | Burial 13 | CNA-1628.1.1 | -18.1 | 10.4 | 19 | 94±61 | 7976 | 33 | 8947-8592 | This study |
| **Casa Corona** | Skeleton 2 | Oxa-V-2392-92 | -19.5 | 11.6 | 0 | nd | 7116 | 32 | 8007-7866 | [9] |
| **Casa Corona** | Skeleton 1 | Beta-272856 | -19.3 | 8.4 | 0 | nd | 7070 | 40 | 7972-7800 | [9] |
| **Cingle Mas Nou** | Skeleton | Beta-170715 | nd | nd | nd | nd | 6920 | 40 | 7839-7674 | [10] |
| **Cingle Mas Nou** | Skeleton | Beta-170714 | nd | nd | nd | nd | 6910 | 40 | 7835-7670 | [10] |
| **Cingle Mas Nou** | Skeleton | OxA-V-2360-29 | -17.5 | 9.5 | 25 | nd | 6925 | 35 | 7685-7583 | [10] |
| **Cingle Mas Nou** | Skeleton | OxA-V-2360-28 | -18.4 | 7.9 | nd | nd | 6897 | 34 | 7822-7666 | [10] |
| **ATLANTIC FACADE** |  |  |  |  |  |  |  |  |  |  |
| **Cabeço Arruda** | Skeleton 6 | Beta-127451 | -19 | nd | 24 | 140±40 | 7550 | 100 | 8409-8030 | [3] |
| **Cabeço da Amoreira** | CAM-00-01 | TO-11819-R | -16.3 | nd | 50 | 140±40 | 7300 | 80 | 8030-7680 | [4] |
| **Moita de Sebastiao** | skeleton 22 | TO-131 | -16.1 | 12.2 | 51 | 140±40 | 7240 | 70 | 7949-7676 | [3] |
| **Amoreiras** | Skeleton 5 | Beta-125110 | -20.8 | nd | 0 | 250±25 | 7230 | 40 | 8160-7970 | [3] |
| **Moita de Sebastiao** | Skeleton 29 | TO-133 | -16.9 | 10.4 | 44 | 140±40 | 7200 | 70 | 7945-7675 | [3] |
| **Arapouco** | Skeleton 2A | Sac-1560 | -16.9 | nd | 44 | 250±25 | 7200 | 130 | 8049-7572 | [15] |
| **Moita de Sebastiao** | skeleton 24 | TO-132 | -16.8 | 11.9 | 45 | 140±40 | 7180 | 70 | 7936-7661 | [3] |
| **Moita de Sebastiao** | skeleton 41 | TO-134 | -16.7 | 11.2 | 46 | 140±40 | 7160 | 80 | 7854-7670 | [3] |
| **Cova da Onça** | unknown | Beta-127448 | -17.2 | nd | 41 | 140±40 | 7140 | 40 | 7856-7674 | [3] |
| **Cabeço da Amoreira** | Burial 2011.1 | Wk-32143 | -15,9 | 13,86 | 56 | 140±40 | 7132 | 41 | 7784-7594 | [4] |
| **Moita de Sebastiao** | skeleton 16 | Beta-127449 | -16.8 | nd | 45 | 140±40 | 7120 | 40 | 7831-7647 | [3] |
| **Cabeço Arruda** | base | TO-10216 | -17.9 | 10.6 | 34 | 140±40 | 7040 | 60 | 7832-7594 | [3] |
| **Cabeço Arruda** | Skeleton 3 | TO-360 | -17.7 | 11.2 | 26 | 140±40 | 6990 | 110 | 7933-7515 | [3] |
| **Cabeço Arruda** | Skeleton A | TO-354 | -19.0 | 12.2 | 24 | 140±40 | 6970 | 60 | 7783-7607 | [3] |
| **Cabeço Arruda** | Skeleton 42 | TO-359a | -17.2 | 11.8 | 42 | 140±40 | 6960 | 60 | 7697-7487 | [3] |
| **Cabeço da Amoreira** | Skeleton 7 | Beta-127450 | -16.5 | 11.9 | 48 | 140±40 | 6850 | 40 | 7564-7429 | [4] |
| **Moita de Sebastiao** | Skeleton CT | TO-135 | -15.3 | 13.4 | 59 | 140±40 | 6810 | 70 | 7492-7312 | [3] |
| **Cabeço Arruda** | Skeleton D | TO-355 | -18.9 | 10.3 | 25 | 140±40 | 6780 | 80 | 7658-7425 | [3] |
| **Cabeço do Pez** | Skeleton 4 | Beta-125109 | -22.6 | nd | 0 | 250±25 | 6760 | 40 | 7703-7580 | [6] |
| **Cabeço do Pez** | Skeleton 4 | Sac-1558 | -19.3 | nd | 0 | 250±25 | 6740 | 110 | 7820-7428 | [6] |
| **Cabeço da Amoreira** | Burial CAM-01-01 | TO-10218 | -17.1 | nd | 42 | 140±40 | 6630 | 60 | 7435-7210 | [4] |
| **Cabeço Arruda** | Top | TO-10217 | -18.1 | 10.5 | 32 | 140±40 | 6620 | 60 | 7466-7261 | [3] |
| **Cabeço da Amoreira** | Burial CAM-01-01 | TO-10225 | -20.1 | 8.2 | 0 | 140±40 | 6550 | 70 | 7572-7324 | [4] |
| **Samouqueira** | Burial H2 | TO-130 | -15.3 | 16.5 | 59 | 250±25 | 6370 | 70 | 7083-6790 | [6] |
| **Cabeço Arruda** | Skeleton N | TO-356 | -15.3 | 12.5 | 59 | 140±40 | 6360 | 80 | 7156-6733 | [3] |
| **Cabeço da Amoreira** | Burial CAM-01-01 | Wk-26796 | -16.9 | 12.3 | 46 | 140±40 | 6329 | 40 | 7145-6858 | [4] |
| **Cabeço de Amoreira** | Burial 2011,2 | Wk-32142 | -15.7 | 12.8 | 58 | 140±40 | 6910 | 40 | 7566-7429 | [4] |
| **CANTABRIAN FACADE** | | | | | | | | | | |
| **Braña/Arintero** | 2 | Beta-226473 | -18.9 | 10.6 | 0 | nd | 7030 | 50 | 7959-7745 | [8] |
| **Braña/Arintero** | 1 | Beta-226472 | -19.3 | 10.6 | 0 | nd | 6980 | 50 | 7932-7696 | [8] |
| **Canes** | 6I | AA-5294 | -20.0 | 7.87 | 0 | nd | 6265 | 75 | 7413-6970 | [8] |
| **Canes** | 6I | OxA-7148 | nd | nd | nd | nd | 6160 | 55 | 7239-6898 | [8] |
| **Canes** | 6II | AA-5296 | -19.7 | 8.5 | 0 | nd | 6770 | 65 | 7736-7505 | [8] |
| **Canes** | 6II | AA-5295 | -19.2 | 9.3 | 0 | nd | 6860 | 65 | 7834-7586 | [8] |
| **Canes** | 6II | AA-11744 | -19.6 | 7.85 | 0 | nd | 7025 | 80 | 7981-7686 | [8] |
| **Canes** | 6III | AA_6071 | -20.9 | 9.5 | 0 | nd | 6930 | 95 | 7944-7609 | [8] |
| **Canes** | 7 | TO-11219 | -21 | 7.7 | 0 | nd | 5980 | 80 | 7151-6636 | [8] |
