# Supplementary material for: The Emergence of Mesolithic Cemeteries in SW Europe: Insights from the El Collado (Oliva, Valencia, Spain) Radiocarbon Record
Source: PLoS One. 2015 Jan 28;10(1):e0115505. doi: 10.1371/journal.pone.0115505 (PMC4309619; doi:10.1371/journal.pone.0115505)
Supplement: S1 Text — (DOC) [file pone.0115505.s002.doc]

**Comments supplementary materials**

The radiocarbon record of Iberian Mesolithic human remains has been the subject of a recent review to homogenize and make comprehensible a highly diverse body of paleoanthropological and contextual data [1–2]. We refer to both works for further details on contextual information about the sites cited below. As mentioned in the introductory section, because our aim is to focus on the chronology of the Iberian cemeteries, we only consider sites with two or more burials in anatomical arrangement.

The radiocarbon record of the Iberian sites meeting such a criterion is presented in Table 4. In order to conduct inter-regional comparisons on the emergence of cemetery-funerary phenomenon, the radiocarbon dates have been grouped in three regional domains: Mediterranean, Atlantic and Cantabrian (Table 1 of supplementary materials).

Naturally, the dataset is quantitatively heterogeneous. A representative sample of dated human skeletons has been obtained at the main Mesolithic sites on the Muge river (Cabeço da Arruda n=7, Cabeço da Amoreira n=7, and Moita do Sebastiao=6) [3–5].

The number of human burials dated in the Sado valley and the Alentejo is quantitatively discreet. Even though the Late Mesolithic sites of Arapouco, Amoreiras, Cabeço do Pez sites and Samouqueira have provided more than one burial [1, 6–7], only one individual from each of those sites has been radiometrically dated.

For the Cantabrian region, just two sites -Canes and Braña Arintero- meet the criteria specified above. In Canes, there are radiocarbon dates for four different individuals, and in Braña for the two individual graves [8].

Finally, in the Mediterranean region, the number of radiocarbon dates for human skeletons per cemetery is as follows: El Collado n=10, Cingle del Mas Nou=4 and Casa Corona n=2 [9–10 and this study]. We will omit the conventional radiocarbon determinations previously published for Burials 4, 6 and 13 at El Collado [11] since those skeletons have been re-dated in the present study.

For each radiocarbon sample, we have detailed the site name, the skeleton or burial identifier, the radiocarbon laboratory code, the BP age and the standard deviation. In addition, when available, δ13C and δ15N stable isotope values and the % of marine diet are provided. Data quality control values associated with each sample (yield, %N, %C, and C:N) have only been published in detail for the Mediterranean cemeteries, not for the remaining Iberian regions [9–10, 12 and this study]. We refer to these published works and, in turn, we assume that the radiocarbon samples for the Cantabrian and the Atlantic regions reported here meet the quality criteria to be considered valid.

The 2σ cal BP ranges are given in the right-hand column. As in the case of El Collado dataset, each radiocarbon date conducted on samples with a significant marine diet contribution has been calibrated using an Intcal 13 mixed marine-terrestrial calibration curve, considering different local ΔR vales.

For the Atlantic facade we used two different local ΔR values. For those sites located in the Muge valley (Cabeço Arruda, Cabeço da Amoreira, Moita do Sebastiao and Cova da Onça), we followed the local 140±40 ΔR value [3, 13]. For the remaining Atlantic sites (Amoreiras, Cabeço do Pez, Samouqueira and Arapouco), all of them located in the Sado valley in the Alentejo province, we used the present day 250±25 ΔR value for the Portuguese coast [14].

For Cantabrian Spain, all human remains considered here have a mainly C3 terrestrial diet [8]. Therefore, they were calibrated just using an atmospheric curve.

For the Mediterranean samples, we have considered the regional ΔR value of 94±61. In addition to El Collado, radiocarbon dates and associated stable isotope values from two new Mesolithic cemeteries have recently been published [9–10]. Palaeodietary evidence at both sites indicates a prevalent C3 terrestrial diet for most of the individuals analyzed. At Cingle del Mas Nou site, one individual (Lab. Code OxA-V-2360-29) aged 3-5 yrs yielded δ^13^C and δ^15^N values showing a marine dietary input comparable to that documented in the individuals 3 and 4 at El Collado. Even though in the stable isotope study of Cingle del Mas Nou the percentage of marine diet is not calculated [10], for radiocarbon calibration purposes we have assumed a 25% marine diet on the basis of the similarities with the isotopic values of individuals 3 and 4 from El Collado.

**References**

1. Meiklejohn C (2009) Radiocarbon dating of the Mesolithic human remains in Spain. Mesolithic Miscellany 20 (2): 2-20

2. Meiklejohn C, Roksandic M, Jackes M, Lubell D (2009) Radiocarbon dating of the Mesolithic human remains in Portugal. Mesolithic Miscellany 20 (1): 2-16.

3. Bicho N, Umbelino U, Detry C, Pereira T (2010) The emergence of the Muge Mesolithic shellmiddens (central Portugal) and the 8200 cal yr BP cold event. Journal of Island and Coastal Archaeology 5: 86-104.

4. Bicho N, Cascalheira J, Marreiros J, Gonçalves C, Pereira T, et al. (2013) Chronology of the Mesolithic occupation of the Muge valley, central Portugal: The case of Cabeço da Amoreira. Quaternary International 308-309: 130-139.

5. Roksandic M (2006) Analysis of burials from new excavations of the sites Cabeço da Amoreira and Cabeço da Arruda (Muge, Portugal). In: Bicho N, Veríssimo H, editors. Do Epipaleolitico ao Calcolítico na Peninsula Iberica. Actas do IV Congresso de Arqueologia Peninsular, Universidade do algarve, Faro: 43-54.

6. Diniz M, Arias P (2012) O Povoamento humano do paleo-estuário do Sado (Portugal): Problemáticas em torno da ocupaçao dos concheiros mesolíticos. In: Campar-Almeida A, Bettencourt AMS, Moura D, Monteiro-Rodrigues S, Caetano-Alves MI, editors. Environmental Changes And Human Interaction Along The Western Atlantic Edge mudanças Ambientais E Interação Humana Na Fachada Atlântica Ocidental, Coimbra: 139-157.

7. Lubell D, Jackes M, Sheppard P, Rowley-Conwy P (2007) The Mesolithic-Neolithic in the Alentejo: archaeological investigations, 1984-1986.  In: Bicho N, editor. From the Mediterranean basin to the Portuguese Atlantic shore: Papers in Honor of Anthony Marks.  Actas do IV Congresso de Arqueologia Peninsular.  Centro do Estudos de Patrimonio, Departamento de Historia, Arqueologia et Patrimonio, Universidade do Algarve: Faro: 209-229.

8. Arias P, Schulting R (2011) Análisis de los isótopos estables de los restos humanos de La Braña-Arintero. Aproximación a la dieta de los grupos mesolíticos de la cordillera Cantábrica. In: Vidal JM, Prada ME, editors. Los hombres mesolíticos de la Cueva de la Braña-Arintero (Valdelugueros, León). Estudios y Catálogos de la Junta de Castilla y León 18, León: 130-137.

9. Fernández-López de Pablo J, Salazar D, Subirà ME, Roca C, Gómez M, et al. (2013) Late Mesolithic burials at Casa Corona (Villena, Spain): direct radiocarbon and palaeodietary evidence of the last forager populations in Eastern Iberia. Journal of Archaeological Science 40: 671-680.

10. Salazar-García DC, Emili-Aura J, Olària CR, Talamo S, Morales JV, et al. (2014) Isotope evidence for the use of marine resources in the Eastern Iberian Mesolithic. Journal of Archaeological Science 42: 231-240.

11. Aparicio J (2008) La necrópolis mesolítica de El Collado (Oliva-Valencia). Varia VIII. Diputación provincial de Valencia.

12. García-Guixé E, Richards MP, Subirà ME (2006) Paleodiets of humans and fauna at the Spanish Mesolithic site of El Collado. Current Anthropology 47: 549-557

13. Martins J, Carvalho A, Soares A (2008) A calibração das datas de radiocarbono dos esqueletos humanos de Muge. Promontoria 6: 73-94.

14. Soares A, Dias A (2006) Coastal upwelling and radiocarbon. Evidence for temporal fluctuations in ocean reservoir effect during the Holocene. Radiocarbon 48(1): 46-60.

15. Cunha E, Umbelino C (2001) Mesolithic people from Portugal: An approach to Sado osteological series. Anthropologie 39: 125–132.
